# SENIOR CENTER INVOLVEMENT WITH DEMENTIA-FRIENDLY COMMUNITIES: COMMUNITY AND ORGANIZATIONAL FACTORS

**DOI:** 10.1093/geroni/igac059.091

**Published:** 2022-12-20

**Authors:** Ceara Somerville, Clara Scher, Caitlin Coyle, Emily Greenfield, Ayse Akincigil

**Affiliations:** University of Massachusetts Boston, Boston, Massachusetts, United States; Rutgers, The State University of New Jersey, New Brunswick, New Jersey, United States; University of Massachusetts Boston, Boston, Massachusetts, United States; Rutgers, The State University of New Jersey, New Brunswick, New Jersey, United States; Rutgers, The State University of New Jersey, New Brunswick, New Jersey, United States

## Abstract

As local hubs for aging services, senior centers are well-positioned to engage in dementia-friendly community (DFC) work. Yet centers vary in their engagement, especially as the DFC concept has been introduced only recently in the US. Using a mixed-methods approach, we drew on data from a survey of senior centers in Massachusetts, the US Census, and qualitative interviews with senior center staff to examine factors associated with DFC engagement. Centers that reported greater engagement were in municipalities with higher proportions of older residents from vulnerable groups (e.g., adults ages 80+, limited English proficiency, with a disability, living alone). They also reported greater programmatic, social service, funding, and staff capacity. Qualitative findings elucidated how senior center leaders drew on intrapersonal, interpersonal, organizational, and community assets to support local DFC efforts. We discuss implications for policies and practices to cultivate senior centers and other community-based organizations as leaders and partners toward DFCs.

